# Molecular monitoring by *CDKN2A/p16^INK4A^* and *RB1* gene methylation in breast cancer

**DOI:** 10.1590/1806-9282.20231358

**Published:** 2024-05-03

**Authors:** Luiz Fernando de Queiroz, Marcelo Soares da Mota e Silva, Fernando Colonna Rosman, Siane Lopes Bittencourt Rosas, Heitor Siffert Pereira de Souza, Maria da Glória da Costa Carvalho

**Affiliations:** 1 Universidade Federal do Rio de Janeiro, Faculty of Medicine, Postgraduate Program in Pathological Anatomy, Department of Pathology – Rio de Janeiro (RJ), Brazil.; 2 Universidade Federal do Rio de Janeiro, Faculty of Medicine, Department of Pathology – Rio de Janeiro (RJ), Brazil.; 3 Universidade Federal do Rio de Janeiro, Faculty of Medicine, Department of Clinical Medicine – Rio de Janeiro (RJ), Brazil.

**Keywords:** Retinoblastoma, Breast neoplasms, DNA methylation

## Abstract

**OBJECTIVE:**

This prospective study aimed to provide a comprehensive analysis of the methylation status of two pivotal genes, *CDKN2A*/*p16* ^INK4A^ (cyclin-dependent kinase inhibitor 2A) and *RB1* (retinoblastoma transcriptional corepressor 1), in breast cancer patients.

**METHODS:**

Samples were obtained from 15 women diagnosed with breast cancer and who underwent a total mastectomy. DNA was extracted from the tumor, non-tumor tissue, and peripheral blood (circulating cell-free DNA). The methylation pattern of cell-free DNA extracted from blood collected on the day of mastectomy was compared with the methylation pattern of cell-free DNA from blood collected 1 year post-surgery. The methylation analysis was carried out by sodium bisulfite conversion and polymerase chain reaction, followed by electrophoresis.

**RESULTS:**

Methylation of *CDKN2A*/*p16* ^INK4A^ was identified in 13 tumor samples and 12 non-tumor tissue samples. Two patients exhibited *CDKN2A*/*p16* ^INK4A^ methylation in the cell-free DNA of the first blood collection, while another showed methylation only in the cell-free DNA of the subsequent blood collection. Regarding *RB1*, 11 tumors and 8 non-tumor tissue samples presented methylation of the gene.

**CONCLUSION:**

This study presents a novel approach for monitoring breast cancer patients through the analysis of cell-free DNA methylation. This analysis can detect changes in methylation patterns before any visible sign of cancer appears in breast tissue and could help predict the recurrence of malignant breast tumors.

## INTRODUCTION

Activation of oncogenes and inactivation of tumor suppressor genes are genetic oscillations related to cancer development^1,2^. DNA methylation, which is an epigenetic mechanism, can influence the expression of tumor suppressors and genes related to uncontrolled cell proliferation. The mechanism can inactivate gene expression and consequently prevent protein synthesis.

The *CDKN2A*/*p16* ^INK4A^ (cyclin-dependent kinase inhibitor 2A), located at chromosome 9 (9p21.3), is a tumor suppressor gene that encodes *p16* ^INK4A^, a protein involved in cell cycle regulation^1,3,4^. The *p16* ^INK4A^ participates in the G1-to-S-phase checkpoint and may interrupt the cell cycle in response to various stressors, leading to the inhibition of cell proliferation^1,5^. Additionally, p16INK4A promotes apoptosis of tumor cells and can increase sensitivity to chemotherapy in breast cancer^3^. An investigation found a positive correlation between hypermethylation of *CDKN2A*/*p16* ^INK4A^ and breast cancer progression and also verified that *CDKN2A*/*p16* ^INK4A^ hypermethylation impacts the tumor's grade and stage^3^.

The *RB1* (retinoblastoma transcriptional corepressor 1) is also a tumor suppressor gene and is located on chromosome 13 (13q14.2). *RB1* encodes the retinoblastoma protein (Rb), and alterations in its tumor suppressor pathway can be considered a potential risk for the development of breast cancer^6–8^. *RB1* can be inactivated by several mechanisms, including alterations in phosphorylation, viral oncoproteins, and promoter hypermethylation^7^.

The analysis of methylation may be performed in circulating cell-free DNA (cfDNA) using liquid biopsy, a promising method for early detection and monitoring of breast cancer^9^. Liquid biopsy is non-invasive and easy to perform, and analysis of cfDNA has a hopeful potential for the study of cancer biomarkers, overcoming difficulties in obtaining and repeating biopsies of metastatic tissues^9–12^.

This study analyzed the methylation statuses of *CDKN2A*/*p16* ^INK4A^ and *RB1* genes in the tumor, non-tumor tissue, and cfDNA of breast cancer patients. The methylation was monitored in cfDNA at two different time points: the day of mastectomy and 1 year post-surgery.

## METHODS

### Study design

This prospective study included 15 women aged between 44 and 78 years (mean age 56.7±9.6 years) diagnosed with breast carcinoma. The patients were treated at the “Instituto de Ginecologia” of “Universidade Federal do Rio de Janeiro,” Brazil, between October 2018 and July 2021. All patients underwent a total mastectomy of one breast. The patients were interviewed and invited to take part in the study. After receiving all the necessary information, those who agreed to participate signed the consent form. The number of patients enrolled is smaller than the ideal due to limitations in the availability of women operated on during the study period.

### Data collection and ethical aspects

The recruitment occurred between October 2018 and July 2021. The medical records served as the basis for obtaining clinical and demographic data. The institutional ethics committee approved the study protocol (certificate: CAAE n° 91406118.6.0000.5257 from August 29, 2018).

### Material collection

After pre-mastectomy, samples of the tumor, non-tumor tissue, and 5 mL of peripheral blood were collected immediately for DNA analysis. Approximately 1 year after the surgery, patients were invited for a new blood collection, and a second cfDNA analysis was performed.

### Extraction of DNA from the tumor, surrounding tissue, and blood serum

The DNA extraction from tumor and non-tumor tissues was performed by the phenol:chloroform method^13^, using the Ultra Pure™ Phenol:Chloroform:Isoamyl Alcohol, from Invitrogen™, Cat. No. 15593-031. Quick-gDNA™ MiniPrep Kit (Zymo Research) Cat. No. D3024 was used for cfDNA extraction from blood serum according to the manufacturer's protocol.

### Methylation mechanism

Sodium bisulfite conversion and MSP (methylation-specific polymerase chain reaction (PCR)) were adopted to analyze DNA methylation using the EZ DNA Methylation-Gold TM Kit, Cat. No: D5005, Zymo Research, according to the manufacturer's protocol.

### Polymerase chain reaction

The DNA integrity was confirmed through amplification of exon 5 of the *p53* gene as previously described^14^. For the *CDKN2*/*p16* ^INK4A^ amplification, two pairs of primers were used as follows: *CDKN2A*/*p16* ^INK4A^-U (unmethylated) forward, 5′-TATTAGAGGGTGGGGTGGATTGT-3′ and *CDKN2A*/*p16* ^INK4A^-U reverse, 5′-CAACCCCAAACCACAACCATAA-3′ producing a fragment of 151 base pairs and *CDKN2A*/*p16* ^INK4A^-M (methylated) forward, 5′-TTATTAGAGGGTGGGGCGGATCGC-3′ and *CDKN2A*/*p16* ^INK4A^-M reverse, 5′-ACCCCGAACCGCGACCGTAA-3′ producing a fragment of 150 base pairs^15^. The polymerase used for the MSP was the GoTaq G2 Hot Start Green Master Mix, Cat. No: M7422, Promega. PCR conditions were as follows: initial denaturation at 96°C for 7 min, followed by 35 cycles of 95°C for 1 min, 60°C for 1 min, and 72°C for 1 min. The final extension was performed at 72°C for 7 min. *RB1*-U (unmethylated) forward, 5′-GGGAGTTTTGTGGATGTGAT-3′ and *RB1*-U reverse, 5′-ACATCAAAACACACCCCA-3′ producing a fragment of 172 base pairs and *RB1*-M (methylated) forward, 5′-GGGAGTTTCGCGGACGTGAC-3′ and *RB1*-M reverse, 5′-ACGTCGAAACACGCCCCG-3′ producing a fragment of 172 base pairs^16^. The polymerase used for the MSP was the GoTaq G2 Hot Start Green Master Mix, Cat. No: M7422, Promega. PCR conditions were as follows: initial denaturation at 96°C for 7 min, followed by 35 cycles of 95°C for 1 min, 55°C for 1 min, and 72°C for 1 min. The final extension was performed at 72°C for 5 min.

### Gel electrophoresis and staining

After amplification, PCR products were separated on polyacrylamide gels at a concentration of 10%. Each electrophoretic run had the addition of a negative control and a DNA marker. Gels were stained by the silver nitrate method, allowing visualization of the DNA bands as previously described^17^. In short, DNA fixation with methanol and acetic acid occurred in the first step, followed by impregnation with silver nitrate in the next step and, finally, with sodium hydroxide (NaOH) and formaldehyde to reveal the DNA bands.

## RESULTS

**Figure 1:**
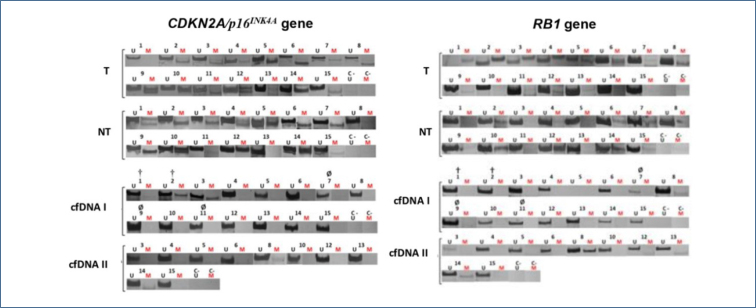
Methylation of *CDKN2A*/*p16* ^INK4A^ and *RB1* genes in breast cancer patients. *CDKN2A*/*p16* ^INK4A^: cyclin-dependent kinase inhibitor 2A; *RB1*: retinoblastoma transcriptional corepressor 1; T: tumor; NT: non-tumor tissue; cfDNA I: circulating cell-free DNA of the first blood collection; cfDNA II: circulating cell-free DNA of the second blood collection; M: methylated DNA; U: unmethylated DNA; C–: negative control; †: death; Ø: no return to the second blood collection for other reason than death. Numbers correspond to patients.

According to the data obtained from medical records, all patients had advanced disease (stage III or IV). Figure 1 shows the methylation statuses of *CDKN2A*/*p16* ^INK4A^ and *RB1* in the tumor, non-tumor tissue, and cfDNA (from the first and second blood collections). *CDKN2A*/*p16* ^INK4A^ methylation was detected in 13 tumors and 12 non-tumor tissue samples. Only two patients (2 and 3) presented *CDKN2A*/*p16* ^INK4A^ methylation in the cfDNA from the blood of the first collection, and only patient 14 presented it in the cfDNA from the blood of the second collection. Furthermore, *RB1* methylation was detected in 11 tumors and 8 non-tumor tissues. Patient 8 showed a weak band of *RB1* methylation in the cfDNA from the blood of the first collection and a strong band of methylation in the cfDNA from the blood of the second collection. Moreover, patient 14 showed a weak band of methylation only in the cfDNA from the second collection.

Tables 1 and 2 present the methylation panels of *CDKN2A*/*p16* ^INK4A^ and *RB1*, respectively.

**Table 1:** Methylation panel of *CDKN2A*/*p16* ^INK4A^ gene in the tumor, non-tumor tissue, and cell-free DNA of breast cancer patients.

Patient number	Tumor	Non-tumor tissue	cfDNA first collection	cfDNA second collection	NC
1	U	M	U	Death	Yes
2	M	M	M	Death	No
3	M	M	M	U	Yes
4	M	M	U	U	Yes
5	M	M	U	U	Yes
6	M	M	U	U	Yes
7	M	M	U	NR	Yes
8	M	U	U	U	Yes
9	M	M	U	NR	No
10	M	M	U	U	No
11	M	U	U	NR	Yes
12	M	M	U	U	Yes
13	U	U	U	U	Yes
14	M	M	U	M	No
15	M	M	U	U	Yes

*CDKN2A*/*p16* ^INK4A^: cylclin-dependent kinase inhibitor 2A; cfDNA: circulating cell-free DNA; M: methylated; U: unmethylated; NR: no return to second blood collection for reason other than death; NC: neochemotherapy.

**Table 2:** Methylation panel of *RB1* gene in the tumor, non-tumor tissue, and cell-free DNA of breast cancer patients.

Patient number	Tumor	Non-tumor tissue	cfDNA first collection	cfDNA second collection	NC
1	M	U	U	Death	Yes
2	M	M	U	Death	No
3	M	U	U	U	Yes
4	M	U	U	U	Yes
5	M	U	U	U	Yes
6	M	M	U	U	Yes
7	M	M	U	NR	Yes
8	M	M	M	M	Yes
9	M	U	U	NR	No
10	U	M	U	U	No
11	M	U	U	NR	Yes
12	M	M	U	U	Yes
13	U	U	U	U	Yes
14	U	M	U	M	No
15	U	M	U	U	Yes

*RB1*: retinoblastoma transcriptional corepressor 1; cfDNA: circulating free DNA; M: methylated; U: unmethylated; NR: no return to second blood collection for reason other than death; NC: neochemotherapy.

## DISCUSSION

This study investigated the methylation statuses of *CDKN2A*/*p16* ^INK4A^ and *RB1* genes in breast cancer patients. DNA from the tumor, non-tumor tissue, and blood serum (cfDNA) was analyzed. The analyses showed that the vast majority of tumor samples had methylation of both *CDKN2A*/*p16* ^INK4A^ (13/15) and *RB1* (11/15). This result was expected as methylation of these genes is related to breast cancer development, as pointed out by Cheng et al.^3^ and Yao^7^. Regarding the non-tumor tissue, the majority of samples (although slightly less than in tumors) also showed methylation of *CDKN2A*/*p16* ^INK4A^ (12/15) and *RB1* (8/15). This confirms that non-tumor tissue, although apparently free of malignancy, is part of the tumor microenvironment. These changes were not detected by histopathological examination, but the molecular analysis of methylation indicated that the tissue considered tumor-free was already in the process of molecular modification with potential for future cancerization.

Only a small number of cfDNA samples showed methylation of *CDKN2A*/*p16* ^INK4A^ (2 and 3 in the first collection, and none in the second collection) and *RB1* (8 in the first collection and 8 and 14 in the second collection). This must be related to the chemotherapy treatment given to patients before mastectomy. As highlighted by Kujala et al.^11^, chemotherapy reduces the concentration of tumor DNA in the blood.

Patient 14 had no methylation in either *CDKN2A*/*p16* ^INK4A^ or *RB1* in the cfDNA of the first blood collection. In contrast, both genes were methylated in the cfDNA of the second blood collection. In addition, patient 8 showed a weak band of *RB1* methylation in the cfDNA from the blood of the first collection and a strong band in the cfDNA of the second collection. The fact that the bands corresponding to methylation are stronger in the cfDNA from the second blood collection suggests a possible recurrence of the disease. Consequently, an increased concentration of tumoral DNA released into the bloodstream is observed, which is visualized as stronger DNA bands by gel electrophoresis.

## CONCLUSION

This study presented a novel approach for monitoring breast cancer patients through the assessment of methylation in cfDNA. Once liquid biopsy is non-invasive and easy to perform, the long-term follow-up of patients is facilitated. The cfDNA analysis proposed here can detect changes in methylation patterns before any visible sign of disease appears in breast tissue. This suggests that the study of cancer-related gene methylation in cfDNA could help predict the recurrence of malignant breast tumors.

## References

[1] Aftab A, Shahzad S, Hussain HMJ, Khan R, Irum S, Tabassum S (2019). *CDKN2A/P16INK4A* variants association with breast cancer and their in-silico analysis. Breast Cancer.

[2] Kontomanolis EN, Koutras A, Syllaios A, Schizas D, Mastoraki A, Garmpis N (2020). Role of oncogenes and tumor-suppressor genes in carcinogenesis: a review. Anticancer Res.

[3] Cheng T, Wu Y, Liu Z, Yu Y, Sun S, Guo M (2022). CDKN2A-mediated molecular subtypes characterize the hallmarks of tumor microenvironment and guide precision medicine in triple-negative breast cancer. Front Immunol.

[4] Andersson N, Saba KH, Magnusson L, Nilsson J, Karlsson J, Nord KH (2023). Inactivation of *RB1*, *CDKN2A*, and *TP53* have distinct effects on genomic stability at side-by-side comparison in karyotypically normal cells. Genes Chromosomes Cancer.

[5] Inoue K, Fry EA (2018). Aberrant expression of p16INK4a in human cancers – a new biomarker?. Cancer Rep Rev.

[6] Linn P, Kohno S, Sheng J, Kulathunga N, Yu H, Zhang Z (2021). Targeting RB1 loss in cancers. Cancers (Basel).

[7] Yao Y, Gu X, Xu X, Ge S, Jia R (2022). Novel insights into RB1 mutation. Cancer Lett.

[8] Abbasi-Kolli M, Shahbazi S, Geranpayeh L (2023). Down-regulation of RB1 and miR-132 in ductal carcinoma of the breast. Int J Mol Epidemiol Genet.

[9] Vietri MT, D'Elia G, Benincasa G, Ferraro G, Caliendo G, Nicoletti GF (2021). DNA methylation and breast cancer: a way forward (review). Int J Oncol.

[10] Sher G, Salman NA, Khan AQ, Prabhu KS, Raza A, Kulinski M (2022). Epigenetic and breast cancer therapy: promising diagnostic and therapeutic applications. Semin Cancer Biol.

[11] Kujala J, Hartikainen JM, Tengström M, Sironen R, Auvinen P, Kosma VM (2022). Circulating cell-free DNA reflects the clonal evolution of breast cancer tumors. Cancers (Basel).

[12] Main SC, Cescon DW, Bratman SV (2022). Liquid biopsies to predict CDK4/6 inhibitor efficacy and resistance in breast cancer. Cancer Drug Resist.

[13] Maccormick TM, Carvalho CES, Bravo GP, Carvalho MDGDC (2019). Comparative analysis of glutathione transferase genetic polymorphism, *Helicobacter pylori* and Epstein-Barr virus between the tumor area and the proximal and distal resection margins of gastric cancer. Rev Col Bras Cir.

[14] Pestaner JP, Bibbo M, Bobroski L, Seshamma T, Bagasra O (1994). Potential of the in situ polymerase chain reaction in diagnostic cytology. Acta Cytol.

[15] Rosas SL, Koch W, Costa Carvalho MG, Wu L, Califano J, Westra W (2001). Promoter hypermethylation patterns of p16, O6-methylguanine-DNA-methyltransferase, and death-associated protein kinase in tumors and saliva of head and neck cancer patients. Cancer Res.

[16] Sippl C, Urbschat S, Kim YJ, Senger S, Oertel J, Ketter R (2018). Promoter methylation of RB1, P15, P16, and MGMT and their impact on the clinical course of pilocytic astrocytomas. Oncol Lett.

[17] Silva MM, Fonseca CO, Moura R, Carvalho JF, Quirico-Santos T, Carvalho MG (2013). Influence of GSTM1 and GSTT1 polymorphisms on the survival rate of patients with malignant glioma under perillyl alcohol-based therapy. Genet Mol Res.

